# Zika virus promotes CCN1 expression via the CaMKIIα-CREB pathway in astrocytes

**DOI:** 10.1080/21505594.2020.1715189

**Published:** 2020-01-20

**Authors:** Jianhong Sun, Wanpo Zhang, Zhongyuan Tan, Caishang Zheng, Yan Tang, Xianliang Ke, Yuan Zhang, Yan Liu, Penghui Li, Qinxue Hu, Hanzhong Wang, Panyong Mao, Zhenhua Zheng

**Affiliations:** a CAS Key Laboratory of Special Pathogens and Biosafety, Center for Emerging Infectious Diseases, Wuhan Institute of Virology, Chinese Academy of Sciences, Wuhan, China; b College of life sciences and health, Wuhan university of science and technology, Wuhan, China; c College of Veterinary Medicine, Huazhong Agricultural University, Wuhan, China; d State Key Laboratory of Virology, Wuhan Institute of Virology, Chinese Academy of Sciences, Wuhan, P.R. China; e Beijing Institute of Infectious Diseases,Military Hospital of China, Beijing, P.R. China

**Keywords:** Zika virus, connective tissue growth factor/Nephroblastoma overexpressed (CCN) gene family 1(CCN1), ca2+/calmodulin-dependent protein kinase II, cAMP-responsive element-binding protein (CREB), cAMP response element, ns3 protein

## Abstract

Zika virus (ZIKV) infection in the human central nervous system (CNS) causes Guillain–Barre syndrome, cerebellum deformity, and other diseases. Astrocytes are immune response cells in the CNS and an important component of the blood–brain barrier. Consequently, any damage to astrocytes facilitates the spread of ZIKV in the CNS. Connective tissue growth factor/Nephroblastoma overexpressed gene family 1 (CCN1), an important inflammatory factor secreted by astrocytes, is reported to regulate innate immunity and viral infection. However, the mechanism by which astrocyte viral infection affects CCN1 expression remains undefined. In this study, we demonstrate that ZIKV infection up-regulates CCN1 expression in astrocytes, thus promoting intracellular viral replication. Other studies revealed that the cAMP response element (CRE) in the CCN1 promoter is activated by the ZIKV NS3 protein. The cAMP-responsive element-binding protein (CREB), a transacting factor of the CRE, is also activated by NS3 or ZIKV. Furthermore,a specific inhibitor of CREB, i.e. SGC-CBP30, reduced ZIKV-induced CCN1 up-regulation and ZIKV replication. Moreover, co-immunoprecipitation, overexpression, and knockdown studies confirmed that the interaction between NS3 and the regulatory domain of CaMKIIα could activate the CREB pathway, thus resulting in the up-regulation of CCN1 expression and enhancement of virus replication. In conclusion, the findings of our investigations on the NS3-CaMKIIα-CREB-CCN1 pathway provide a foundation for understanding the infection mechanism of ZIKV in the CNS.

## Introduction

Zika virus (ZIKV), an arbovirus of the genus *Flavivirus* within the family*Flaviviridae*, has recently garnered attentionas it can cause cerebellum deformity in newborns [1]. In the brain, ZIKV preferentially infects neural stem cells, astrocytes, oligodendrocyte precursors, and microglia [2]. Astrocytes are important immune response cells in various neurotropic viral infections [3–6]. They respond to central nervous system(CNS) injury and degenerative diseases [7,8]. Considerable evidence suggests that astrocytes areimportant in CNS homeostasis and are typically involved in maintaining, supporting, and nourishing neurons and in interactions with the endothelia to generate and maintain the blood–brain barrier(BBB) [9,10]. The interaction between ZIKV and astrocytes is of interest to researchers [11–16].

CCN1, also called cysteine-rich protein 61 (Cyr61), is a matricellular secreted protein of the cysteine rich 61/connective tissue growth factor/nephroblastoma overexpressed (CCN) gene family. CCN1 is secreted by reactive astrocytes [17]. It is distributed in a dot shape in the cell membrane, extracellular matrix, and in cytoplasmic perinuclear areas where the endoplasmic reticulum is localized at a high density [18–20]. CCN1 regulates multiple cellular functions through integrin receptors and co-receptors, such as heparin sulfate proteoglycans receptors and low-density lipoprotein receptor-related proteins [21]. Additionally, it regulates cell proliferation, adhesion, migration, differentiation, apoptosis, senescence, and gene expression [21]. Recently, CCN1 has been implicated as a pro-inflammatory factor involved in the regulation of inflammatory molecules [22,23]. It is generally known that CCN1 expression is up-regulated in viral or bacterial infection [24–27]. The presence of CCN1 also facilitates virus replication [25]. Therefore, it is important to investigate the role of CCN1 in the replication and dissemination of ZIKV in the CNS.

Hitherto, multiple pathways have been implicated in the regulation of CCN1 expression, including thecAMP-responsive element-binding protein(CREB) [28]. CREB, a nuclear transcription factor, can be activated by various kinases including theCa^2+^/calmodulin-dependent protein kinase II (CaMKII) and can promote the expression of genes containing the cAMP response element (CRE) [29]. CaMKII, a Ca^2+^/calmodulin-dependent Ser/Thr protein kinase, has four isoforms: CaMKIIα, CaMKIIβ, CaMKIIγ, and CaMKIIδ [30]. Anincrease in the intracellular Ca^2+^ concentration leads to the binding of Ca^2+^ ions to calmodulin (CaM), resulting in the activation of CaMKII. Upon activation, this enzyme autophosphorylates Thr286, a process that confers Ca^2+^-independent activity on the kinase and significantly increases its affinity for CaM [31]. In addition, CaMKII-CREB signallingis critical in the regulation of synaptic plasticity [32,33].

To explore the interactions between ZIKV and CCN1 in astrocytes, we first confirm the role of CCN1 in viral replication and reveal the specific mechanisms by which ZIKV regulates CCN1 expression. These findings provide a foundation for understanding the pathogenesis of ZIKV in the CNS.

## Materials and methods

### Cells culture

Vero cells (African Green Monkey kidney) (CCL-81, American Type Culture Collection) were cultured in minimum essential medium (MEM) (Life Technologies) supplemented with 10% fetal bovine serum (FBS) (Gibco). CCF-STTG1 cells (Human Astrocytoma) (ATCC-CRL-1718) were cultured in Roswell Park Memorial Institute 1640 medium (RPMI-1640) (Life Technologies) supplemented with 10% FBS (Gibco). HEK293T cells (ATCC-CRL-3216) were cultured in Dulbecco’s modified Eagle’s medium (DMEM)(Life Technologies) supplemented with 10% FBS (Gibco). All cells were cultured at 37°C in an atmosphere of 5% CO_2_. *Aedes albopictus* C6/36 cells (ATCC-CRL-1660) were cultured in RPMI-1640 supplemented with 10% FBS (Gibco) at 28°C in an atmosphere of 5% CO_2._

### Virus

ZIKV (Zika virus/SZ01/2016/China, GenBank: KU866423.2) was obtained from the Wuhan Institute ofVirology, Chinese Academy of Science [34] and was propagated in C6/36 cells.Viral titer was determined by TCID_50_ assay; 50 µl of virus suspension was added to 450 µl of MEM. A series of 10-fold dilutions up to a concentration of 10^−11^ was prepared with the MEM. Subsequently, 100 µl of each dilution was loaded in quadruplicate into a 96-well plate of Vero cells, followed by 1.5-h incubation for viral absorption. Subsequently, the supernatant containing virus was removed and replaced with fresh MEM. Four to seven days were required to complete the viral infection cycle when no new cytopathogenic effects (CPEs) appeared in the wells. The CPE was observed, and the number of wells associated with the CPE was entered into the Reed & Muench calculation calculator software [35]. UV-ZIKV was prepared by placing samples on ice, 70 cm below a 30-W UV lamp for 30 min.

### Plasmid construction

The cDNAs of human CCN1^WT^ (CCN1) and nonsecretory signal-CCN1^mut^ (NS-CCN1) were obtained from CCF-STTG1 total RNA by RT-PCR using the CCN1 and NS-CCN1 primers, as shown in Table 1. The RT-PCR fragments were cloned into a pcDNA3.1 (+) vector and digested with NheI and BamHI (TaKaRa).

**Table 1.** Oligos used in vector construction and RT-qPCR assay and RNA interference.

PCR products/purpose	Forward premier(5ʹ→3ʹ)	Reverse premier(5ʹ→3ʹ)
CCN1	CTAgctagcATGAGCTCCCGCATCGCCAG	CGCggatccTTAGTCCCTAAATTTGTGAATGTC
NS-CCN1	CTAgctagcATGACCTGCCCCGCTGCCTGC	CGCggatccTTAGTCCCTAAATTTGTGAATGTC
p-2299	CGGggtaccCCCCATCCCCAACCTCCAA	GGAagatctGCGACGAAGACGCCAACAAG
p-2001	CGGggtaccCAAATAGAAAAGACGCCAC	GGAagatctGCGACGAAGACGCCAACAAG
p-895	CGGggtaccGGCTGGAACTAAAGTGGGAACCT	GGAagatctGCGACGAAGACGCCAACAAG
p-409	CGGggtaccCCCTCACGACCCTCCAACTAC	GGAagatctGCGACGAAGACGCCAACAAG
p-40	CGGggtaccCCGCCGGCCCGTATAAAAG	GGAagatctGCGACGAAGACGCCAACAAG
p-CRE-mut-p-2299–68	CGGggtaccCCCCATCCCCAACCTCCAA	AGGCGCCGCGTGTTGCAGGCTCTGTCTGCGCGTTCC
p-CRE-mut-p-59-+191	CTGCAACACGCGGCGCCTCCGCCGGCCCGTATAAAAG	GGAagatctGCGACGAAGACGCCAACAAG
HA-CaMKIIα 1(1–478)	CTAgctagcATGTACCCATACGACGTCCCAGACTACGCTGCCACCATCACCTGCACCC	CCCaagcttTCAGTGGGGCAGGACGGA
HA-CaMKIIα 261 (261–478)	CTAgctagcATGTACCCATACGACGTCCCAGACTACGCTACAGCTGCCGAAGCCCTT	CCCaagcttTCAGTGGGGCAGGACGGA
HA-CaMKIIα 280 (280–478)	CTAgctagcATGTACCCATACGACGTCCCAGACTACGCTTGCATGCACAGACAGGAGA	CCCaagcttTCAGTGGGGCAGGACGGA
HA-CaMKIIα 301 (301–478)	CTAgctagcATGTACCCATACGACGTCCCAGACTACGCTGGAGCCATTCTCACCACG	CCCaagcttTCAGTGGGGCAGGACGGA
HA-CaMKIIα 315 (315–478)	CTAgctagcATGTACCCATACGACGTCCCAGACTACGCTGGAGGGAAGAGTGGGGG	CCCaagcttTCAGTGGGGCAGGACGGA
HA-CaMKIIα 341 (341–478)	CTAgctagcATGTACCCATACGACGTCCCAGACTACGCTGAAGACACCAAAGTGCGG	CCCaagcttTCAGTGGGGCAGGACGGA
si-CaMKIIα targeting	GGACTTCCATCGATTCTAT	
ZIKV detecting	AARTACACATACCARAACAAAGTG	TCCRCTCCCYCTYTGGTCTTG
human-*gapdh q-PCR*	GTCTCCTCTGACTTCAACAGCG	ACCACCCTGTTGCTGTAGCCAA
mouse-*gapdh q-PCR*	AACGACCCCTTCATTGAC	CCACGACATACTCAGCAC
human-*ccn1 q-PCR*	GAATCTACCAAAACGGGGAAAG	GGGTTGTATAGGATGCGAGGCT
mouse-*ccn1 q-PCR*	CAGAATCTACCAAAACGGGGA	GGTTCGGTGCCAAAGACAGG

The expression plasmids of ZIKV nonstructural proteins, including pCAGGS-Flag-NS1, pCAGGS-Flag-NS2A, pCAGGS-Flag-NS2B, pCAGGS-Flag-NS3, pCAGGS-Flag-NS4A, pCAGGS-Flag-NS4B, and pCAGGS-Flag-NS5, were constructed by cloning the corresponding coding region of the viral proteins from ZIKV cDNA and inserting them into the multiple cloning sites of pCAGGS.

To generate luciferase reporter plasmids of the CCN1 promoter with different regulatory elements, different lengths of promoter regions were amplified from the CCF-STTG1 genomic DNA by PCR using the p-2299 (−2299-+191), p-2001 (−2001-+191), p-895 (−895-+191), p-409 (−409-+191), p-40 (−40-+191), and p-CRE-mut (p-CRE-mutant, the full-length CCN1 promoter deleted −67–60 bases) primers, as shown in Table 1. The p-CRE-mut fragment is constructed by overlap PCR. The primers p-CRE-mut-p-2299–68 and p-CRE-mut-59-+191 are used for amplifyingthe PCR products −2299–68 and −59-+191 respectively, and the two fragments are mixed equally for overlap extension of p-CRE-mut fragments lacking −67–60 bases with primers of p-2299.The PCR fragments were cloned into pGL3 luciferase reporter vectors (Promega) and digested with KpnI and BglII (TaKaRa). Subsequently, the cloned fragments were verified by sequencing.

A series of human CaMKIIα mutants were constructed by inserting PCR fragments amplified from human cDNA library into the pcDNA3.1(+) vector and digesting with NheI and HindIII. The fragments and their corresponding PCR primers are listed in Table 1.

### CCF-STTG1 cells transfection

CCF-STTG1 cells were plated in an appropriate culture dish. When the plated cells reached approximately 70–80% confluence, the cells were transfected using Lipofectamine^TM^ 3000 (Invitrogen), according to the manufacturer’s instructions.

For RNA interference (siRNA) transfection, si-CaMKIIα(targeting sequence in Table 1) and nonspecific scrambled siRNA were purchased from China’s RIBOBIO Co., Ltd. The CCF-STTG1 cells were placed into 6-well plates, andtransfection was performed under optimal conditions of 40-60% confluence. Each transfection was performed with 50-nM siRNA using Lipofectamine^TM^ 3000 (Invitrogen), according to the manufacturer’s instructions.

### Virus infection, inhibitor, and antibody treatments

CCF-STTG1 cells were infected with ZIKV at a multiplicity of infection (MOI) of three tissue infections 50% dose (TCID50)/cell (ZIKV group) or sham-treated with RPMI-1640 medium (mock group). For inhibitor experiments, the cells were infected with ZIKV for 2h in the presence of the indicated inhibitor, washed with RPMI-1640 medium, and placed in a medium containing 10% FBS along with fresh inhibitor, SGC-CBP30 (MedChem Express). For antibody neutralization experiments,the cells were infected with ZIKV for 2h, washed with RPMI-1640 medium, and placed in medium with 10% FBS containing anti-CCN1 antibody (Santa Cruz).

### Ethics approval and participation consent

All animal experiments were performed in strict accordance with the regulations described in the Guide for the Care and Use of Laboratory Animals issued by the Ministry of Science and Technology of the People’s Republic of China. All efforts were made to minimize suffering. Animal protocols complied with the Guidelines for Animal Care approved by the Laboratory Animal Care and Use Committee at the Wuhan Institute of Virology, Chinese Academy of Sciences (Wuhan, China) (approval number: WIVA07201704; approval date 1 September 2016).

### Animal experiments

C57BL/6 mice deficient in IFN I and II receptors (AG6 mouse, *ifnagr-/-*) were obtained from the Institute Pasteur of Shanghai, Chinese Academy of Sciences and bred in specific pathogen-free conditions at the Wuhan Institute of Virology. Nine-week-old AG6 mice were intraperitoneally injected with 10^5^ TCID_50_ of ZIKV in 100 µL of phosphate-buffered saline (PBS). Nine 3-week-old AG6 mice fromthe mock group were administered PBS by the same route. The mice were sacrificed on days 2 and 5 post-infection; the whole brain of three mice in each group was dissected and homogenized in 1-mL cold PBS. Subsequently, 50μLof the homogenizing liquid was added to 950μL TRIzol and frozen at −80°C. On day 5 post-infection, three mice in each group were administered 4% paraformaldehyde (PFA) systemic perfusion after anesthesia, and the whole brain was removed for paraffin embedding.

### Immunofluorescence

Brains were embedded in paraffin wax after gradient ethanol dehydration and xylene transparency and, subsequently, sectioned using a microtome. Additionally, 36 h post-infection, the CCF-STTG1 cells were fixed in 4% PFA and permeabilised in PBS containing 0.5% Triton X-100 and 20-mM glycine.

The standard immunofluorescence staining protocol was followed for tissue or cell staining. Primary antibodies included anti-ZIKV envelope (ZIKV-E) proteinantibody (Biofront) (1:200), anti-glial fibrillary acidic protein (GFAP) antibody (1:100) (Proteintech), anti-p-CREB(Ser133) antibody (1:100) (Abcam),and anti-CCN1 antibody (1:100) (Proteintech). All primary antibodies were incubated with the corresponding secondary antibodies. Counterstaining was performed using4, 6-diamidino-2-phenylindole (DAPI).Fluorescent images of the cells were capturedusing a Nikon fluorescence microscope (Tokyo, Japan). Images of the tissue were acquired usinga whole-slide digital panoramic scanner (3D-Histech, Budapest, Hungary).

### Luciferase assays

HEK293T cells were grown to 40–50% confluence in 12-well plates and co-transfected with an expression plasmid, a luciferase reporter plasmid, and pRL-TK (Renilla luciferase, internal control) using ProFection calcium phosphate reagents, according to the manufacturer’s instructions (Promega). Subsequently, 24–48 h post-transfection, the cells were collected and washed once with cold PBS. Passive lysis buffer (Promega) was subsequently added to the cells. After 15 min, supernatants were collected following centrifugation at 12,000 × *g* for 3 min, and relative luciferase activity was measured with the Dual-Luciferase Reporter Assay System according to the manufacturer’s instructions (Promega).

### RNA isolation, cDNA synthesis, and RT-qPCR

The culture supernatant of 300 μL was placed in 700-μL LS TRIzol (Life Technologies), and the RNA in the culture supernatant was extracted according to the instructions. The culture supernatant was removed, 1 mL TRIzol (Life Technologies) was added, and RNA was extracted from the cell, according to the instructions. TRIzolreagent was used to isolatemouse brain total RNA after homogenization. First-strand cDNA was synthesized using PrimeScript™ RT Reagent Kit with gDNA Eraser (Perfect Real Time) (TaKaRa). For RT-qPCR, cDNA derived from 50-ng RNA was amplified in a 20-µL volume using SYBR GreenER qPCR SuperMix for ABI PRISM (Invitrogen). The primers of human-*gapdh*, mouse-*gapdh*, human-*ccn1*, and mouse-*ccn1*, and the ZIKV, shown in Table 1, were used for RT-qPCR. The RT-qPCR conditions were set to 95°C for 3 min, 40 cycles at 95°C for 10 s, and 60°C for 45 s. To evaluate the copy number of ZIKV and CCN1 in the samples, the RT-qPCR threshold cycle value was set according to the standard curve obtained with plasmids containing DNA sequences amplified from the corresponding ZIKV or CCN1 cDNA at serial dilutions ranging from 1 × 10^3^ to 1 × 10^8^copies/mL.

### TAP purification and mass spectrometry

TAP purification assays were performed according to the standard manufacturer’s instruction (Stratagene). In brief, 293T cells were transfected with pNTAP-A-NS3 or pNTAP-A control vector. After 36 h post-transfection, cells were harvested and lysed. ZIKV NS3-host protein complexes were first purified using washed streptavidin resin and then washed calmodulin resin. The protein complexes were eluted by calmodulin elution buffer (CEB) and rotated the tube at 4ºC for 30 min followed by precipitation by trichloroacetic acid (TCA) method. The precipitated proteins were identified by mass spectrometry.

### Co-IP assay

HEK293T cells were co-transfected with pcDNA-HA-CaMKIIα and pCAGGS-Flag-NS3 or pCAGGS-Flag-NS5 using ProFection calcium phosphate reagents (Promega). Subsequently, 24–36 h post-transfection, the cells were collected using western and IP buffer (Beyotime, Jiangsu, China) containing PMSF protease inhibitor according to the manufacturer’s instructions. Whole-cell extract (30 μL) was used as input; the remainder was divided into two equal parts and incubated with the Flag antibody and IgG, separately, at 4°C overnight with gentle rotation. Protein G magnetic beads (Invitrogen) were added into the lysates and incubated for 2–3 h according to the manufacturers’ instructions. Precipitates were washed five times with TBST buffer, and the proteins were eluted by boiling the beads for 5 min in 1× SDS sample buffer. Eluted proteins and input whole-cell extracts were analyzed by western blot.

### Western blot

Cell lysates were prepared using RIPA buffer (Beyotime, Jiangsu, China) supplemented with PMSF protease inhibitor and phosphatase inhibitor cocktail (Roche) according to the manufacturer’s instructions. Equal amounts of cellular protein extracts were diluted in sodium dodecyl sulfate polyacrylamide gel electrophoresis (SDS-PAGE) sample buffer, heated at 100°C for 15 min, and subjected to 12% SDS-PAGE. Proteins were electroblotted onto Immobilon-P polyvinylidene fluoride membranes (Millipore). Subsequently, nonspecific binding sites were blocked in Tris-buffered saline (TBS) containing 5% (*w*/*v*) nonfat milk powder. All antibodies were diluted in 2.5% (*w*/*v*) nonfat milk powder in TBS. The blots were subsequently analyzed by incubation with the indicated antibody against CCN1 (1:1000) (Proteintech), CREB (1:1000) (Abcam), p-CREB (1:5000) (Abcam), CaMKIIα (1:1000) (Santa Cruz), p-CaMKIIα (1:1000) (Santa Cruz), ZIKV-E protein (1:2000) (Biofront), Flag (1:1000), HA (1:1000),or beta-actin (1:1000) (ComWei), followed by an appropriate secondary antibody. After adding the Immobilon western chemiluminescent HRP substrate (Millipore), the blots were visualized and analyzed using the Bio-Rad imaging system.

### Statistical analysis

Each experiment was repeatedthree times. Statistical analyses were performed using the GraphPad Prism 6.0 software (GraphPad Software, San Diego, CA). Data are expressed as mean ± standard deviation (SD). Probability values of less than 0.05, 0.01, and 0.001 were considered statistically significant and marked with *, **, and ***, respectively, in figures.

## Results

### *ZIKV infects astrocytoma CCF-STTG1 cells and astrocytes* in vivo

**Figure 1.**
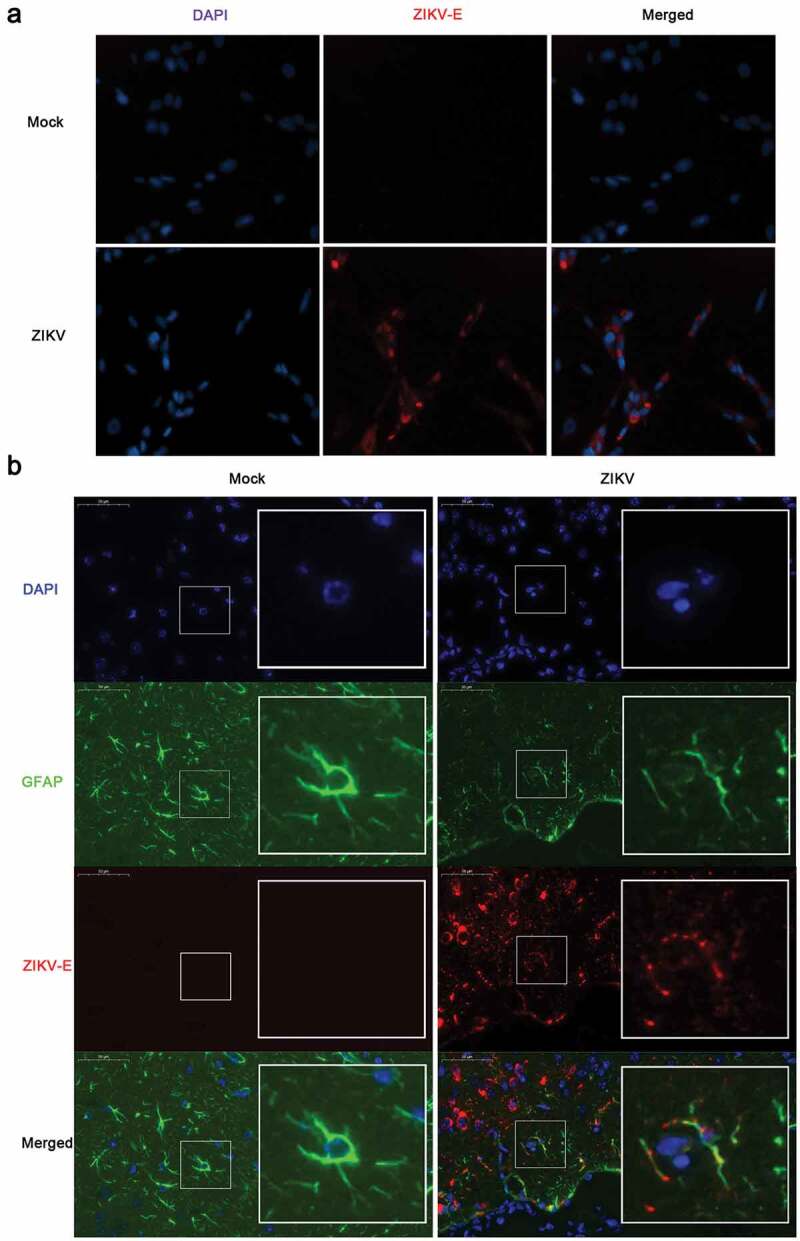
ZIKV infects CCF-STTG1 cells *in vitro* and astrocytes in the brain of AG6 mice *in vivo*. (a) CCF-STTG1 cells were infected with ZIKV at an MOI of 3 (TCID_50_/cell). Subsequently, 24-h post-infection, the cells were used for immunofluorescence analysis. The experiment was repeated three times. ZIKV-E (red), DAPI (blue).10×; (b) Sagittal sections of the brains of AG6 mice on day 5 post-infection. Three mice per group were perfused and paraffin-embedded. GFAP (green), ZIKV-E (red), DAPI (blue).48.4× magnification.(Bar = 50 μm).

To determine whether ZIKV could infect astrocytoma and astrocytes, the following two experiments were performed. CCF-STTG1, a human astrocytoma cell line, was used for *in vitro* studies [36,37]. Immunofluorescence staining demonstrated the presence of ZIKV-Eproteinpositive products in CCF-STTG1 cells (Figure 1(a)).An *in vivo* infection experiment was conducted as well. Nine-week-old AG6 mice were intraperitoneally injected with 10^5^ TCID_50_ of ZIKV. Onday 5 post-infection, the entire brain was perfused with 4% PFA and subsequently stripped and analyzed by immunofluorescence staining. Parts of the astrocytes expressing the characteristic marker, GFAP, were found positive for ZIKV-E protein (Figure 1(b)). Comprehensively, these results indicate that ZIKV can infect astrocytoma *in vitro* and astrocytes *in vivo*, which is consistent with thefindings of previous reports [38,39].

### *ZIKV promotes CCN1 expression in astrocytoma CCF-STTG1 cells and astrocytes* in vivo

**Figure 2.**
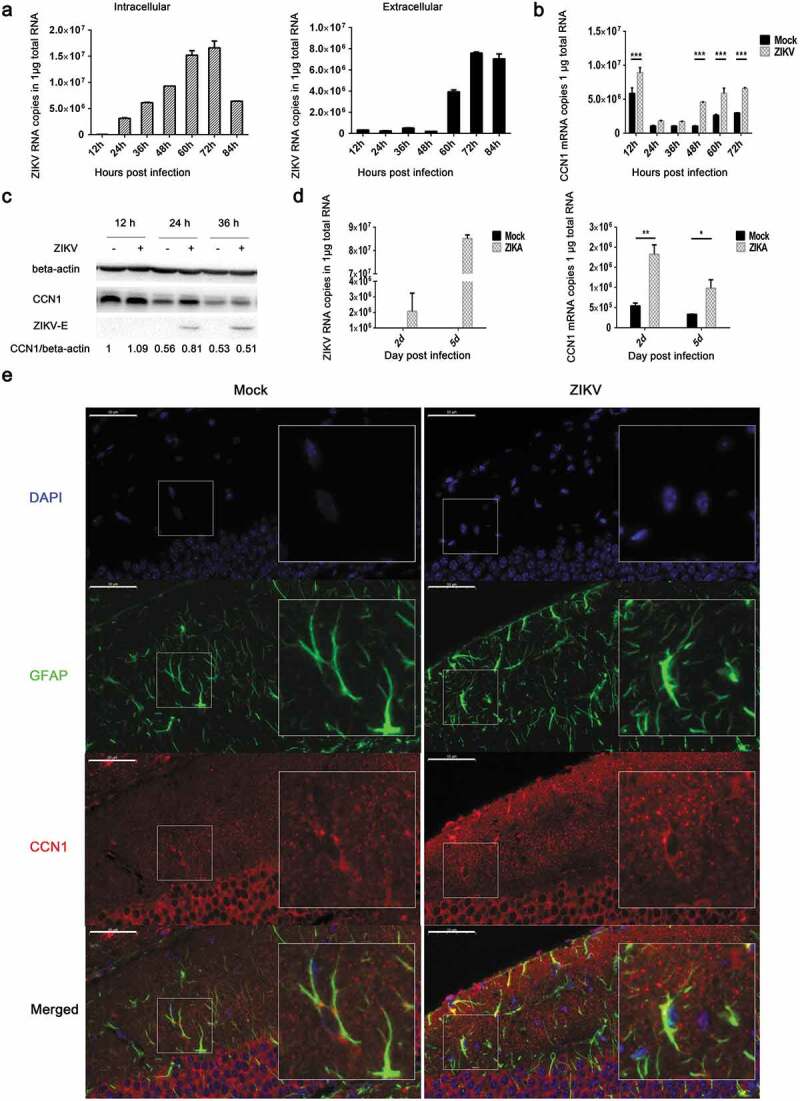
ZIKV promotes CCN1 expression in CCF-STTG1 cells and in mouse brain astrocytes. (a) and (b) MOI = 3TCID_50_/cell. Double standard curve method was used for RT-qPCR. The formula of the standard curve is as follows: human-*gapdh* y = −0.240x + 10.99, human-*ccn1* y = −0.221x + 10.27, ZIKV RNA y = −0.272x + 10.96. (a) Expression profiles of ZIKV RNA in intra- and culture supernatant CCF-STTG1 cells. (b) Expression profiles of CCN1mRNA post-infection in CCF-STTG1 cells. (c) Expression profiles of CCN1 and ZIKV-E protein in CCF-STTG1 cells. (d) Expression profiles of ZIKV RNA and CCN1 mRNA in the brain of mice. On days 2 and 5 post-infection, the whole brain of ZIKV-infected mice and mock-treated mice were stripped and total RNA were extracted and analyzed using RT-qPCR (double standard curve method). The formula of the standard curve is as follows: mouse-*gapdh* y = −0.3025x + 11.048, mouse-*ccn1* y = −0.2234x +10.124, ZIKV RNA y = −0.2721x + 10.966. (e) Immunofluorescence staining of CCN1 and GFAP in the brain of mice. CCN1 (red), GFAP (green), DAPI (blue). (Bar = 50 μm). Each experiment was repeated three times. Statistical analyses were performed using GraphPad Prism 6.0 software. Data are expressed as the mean ± standard deviation (SD) (*, P < 0.05; **, P < 0.01; ***, P < 0.001).

To analyze the expression profile of CCN1 during ZIKV infection in CCF-STTG1 cells and astrocytes, RT-qPCR (double-standard curves method), western blot, and immunofluorescence staining were performed. The cells were infected with ZIKV at an MOI of 3 (TCID_50_/cell).The results indicated that as infection time increased, intracellular viral RNA content also increased inside the cell, peaking at 72h post-infection (Figure 2(a)). In vitro extracellular viral RNA indicated a clear increase at 60h post-infection that also peaked at 72hpost-infection (Figure 2(a)).This proved that the virus was released after 60 h post-infection. Furthermore, these results revealed that ZIKV could replicate and be released in CCF-STTG1 cells. CCN1 mRNA and protein expression levels increased in the ZIKV group compared withthose in the mock group (Figure 2(b,c)). This indicates that the ZIKV infection of human astrocytoma CCF-STTG1 cells *in vitro* up-regulates CCN1 expression. *In vivo*, ZIKV could infect the brain of AG6 mice. To study the effect of ZIKV infection on the expression of CCN1 in mouse brain, the whole brain of mice was stripped and homogenized; RT-qPCR results showed that CCN1 expression at the mRNA levels was up-regulated in the ZIKV group on days 2 and 5 post-infection, compared with that of the mock group (Figure 2(d)). Immunofluorescence staining of brain paraffin sections showed that CCN1 positive products were distributed in the cytoplasm and extracellular matrix of the astrocytes. Compared with those of the mock group, CCN1 positive products in the ZIKV group increased significantly (Figure 2(e)). This indicates that ZIKV infection up-regulated CCN1 expression in mouse astrocytes *in vivo*. Therefore, CCF-STTG1 cells were used as an *in vitro* model to investigate the mechanism underlying ZIKV infection-mediated CCN1 expression in astrocytes.

### CCN1 facilitates ZIKV replication

**Figure 3.**
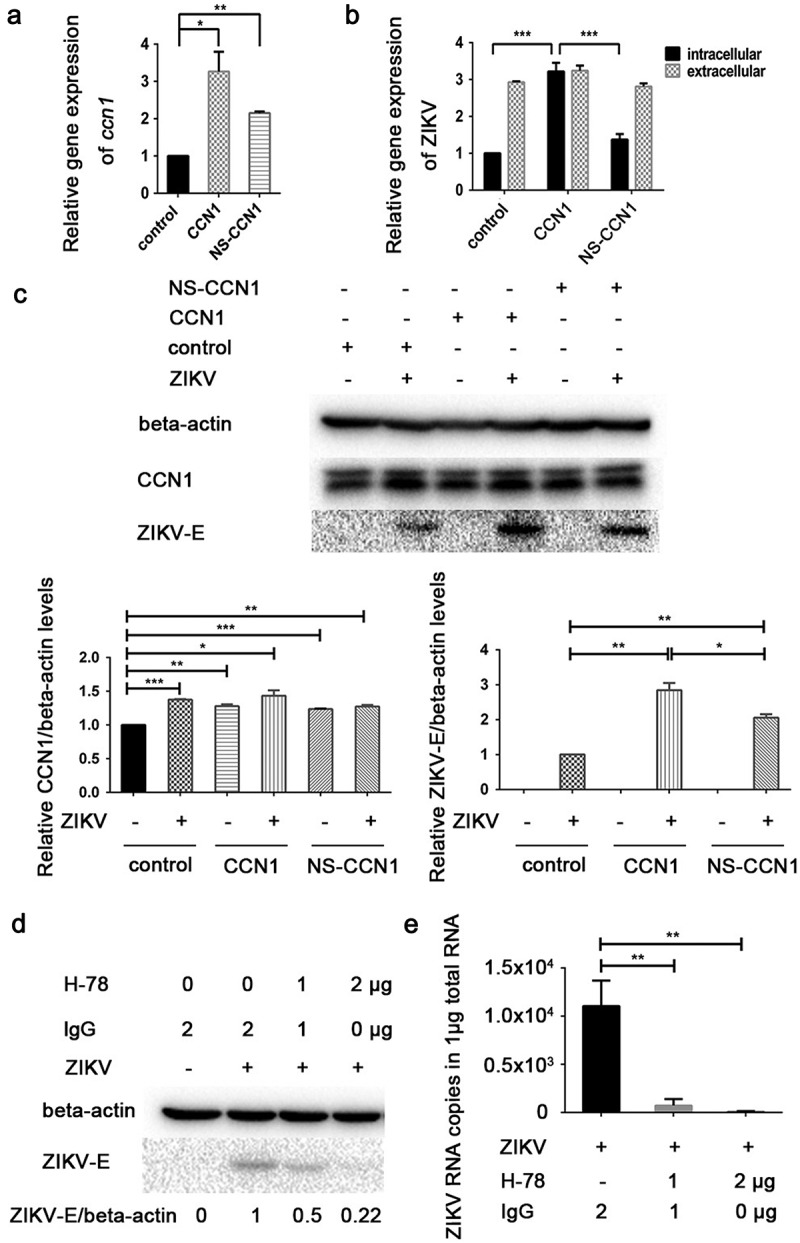
CCN1 promotes ZIKV replication in CCF-STTG1 cells. (A&B) CCN1, NS-CCN1, and pcDNA3.1(+) were transfected in CCF-STTG1 cells, separately. Subsequently, 6 h post-transfection, the cells were infected with ZIKV (MOI = 3TCID_50_/cell). Cells and supernatants were harvested 60 h post-infection. (a) Intracellular CCN1 mRNA levels were measured by RT-qPCR analysis (comparative delta–delta Ct). b) Intracellular and extracellular ZIKV RNA levels were measured by RT-qPCR analysis (comparative delta–delta Ct). (c) CCF-STTG1 cells were transfected with CCN1 or NS-CCN1 expression plasmids, and subsequently infected with ZIKV (MOI = 3 (TCID_50_/cell)), and cell lysates were harvested 24 h post-infection. The CCN1 and ZIKV-E protein level relative to beat actinin three experiments were summarized in the bar graph below. (d)&(e) CCF-STTG1 cells were infected with ZIKV for 2 h, followed by the addition of 1 µg or 2 µg of H-78 in the culture supplied with 1.5-mL complete medium. (d) CCF-STTG1 cells were harvested 36 h post-infection for western blot. Densitometry from ZIKV E blots normalized for beta-actin were labeled below the blots (e) CCF-STTG1 cells were harvested 36 h post-infection for RT-qPCR. Double standard curve method was used for RT-qPCR. The formula of the standard curve is as follows: human-*gapdh* y = −0.438x + 14.02, human-*ccn1* y = −0.262x + 10.90, ZIKV RNA y = −0.243x + 10.42. Each experiment was repeated three times. Statistical analyses were performed using GraphPad Prism 6.0 software. Data are expressed as the mean ± standard deviation (SD) (*, P < 0.05; **, P < 0.01; ***, P < 0.001).

To examine the role of CCN1 in ZIKV infection, CCN1 plasmids, theirnonsecreted mutant NS-CCN1, and the control pcDNA3.1(+) were transfected into CCF-STTG1 cells, separately. The results show that compared with the control group, CCN1 mRNA was highly expressed in CCN1 and NS-CCN1 groups (Figure 3(a)). But,we found that the CCN1 mRNA in NS-CCN1 group was lower than that in CCN1 group(Figure 3(a)). Subsequently, 6 h post-transfection, the cells were infected with ZIKV at an MOI of 3. Toanalyse the effect of CCN1 on viral replication and release, intracellular and extracellular ZIKV RNA were measured by RT-qPCR analysis 60 h post-infection. A significant increase in intracellular viral RNA was detected in the CCN1 group compared with that in the NS-CCN1 and control groups (Figure 3(b)). Extracellular ZIKV RNA levels of the CCN1, NS-CCN1, and control groups exhibited no significant difference (Figure 3(b)). ZIKV-E protein was detected using western blot to investigate the effects of CCN1 on ZIKV infection. CCF-STTG1 cells overexpressing CCN1 or NS-CCN1 were infected with ZIKV at an MOI of 3 (TCID_50_/cell). Subsequently, 24 h post-infection, an increase in ZIKV-E protein expression was detected in the CCN1 group compared with that in the control and NS-CCN1 groups (Figure 3(c)). Of note, compared with the control group, ZIKV-E protein expression in NS-CCN1 groups also showed a small increase (Figure 3(c)). It is suggested that CCN1 may have other intercellular targets on the viral replication.This indicates that CCN1 expression can enhance intracellular ZIKV replication and that CCN1 secretion is important for this process.

CCN1 is a secreted protein. Therefore, we used H-78 (Santa Cruz sc-13,100), a CCN1 antibody, to inhibit the activation of downstream CCN1 signals [40,41]. Following infection with ZIKV for 2 h, 1µg or 2µg of H-78 was added in 1.5 mL of complete medium. CCF-STTG1 cells were harvested 36h post-infection. We found that H-78 blocked the synthesis of ZIKV-E protein in a dose-dependent manner (Figure 3(d)). This suggests that CCN1 downstream signaling is important for the enhancement of ZIKV infection.

### ZIKV NS3 protein activates the CRE in the CCN1 promoter and induces CREB phosphorylation and CCN1 expression

**Figure 4.**
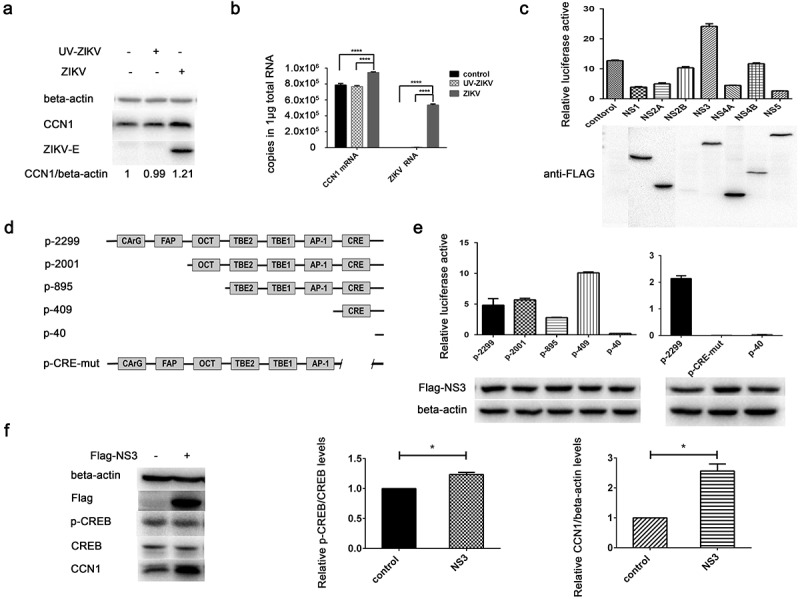
NS3 protein of ZIKV activates the CRE in the CCN1 promoter and induces CREB phosphorylation and CCN1 expression. (a) and (b) CCF-STTG1 cells were infected with ZIKV or UV-ZIKV for 2 h. (a) Cells were harvested 24 h post-infection for western blot analysis. (b) Cells were harvested 12 h post-infection for RT-qPCR analysis. Densitometry from CCN1 blots normalized for beta-actin were labeled below the blots. Double standard curve method was used for RT-qPCR. The formula of the standard curve is as follows: human-*gapdh* y = −0.438x + 14.02, human-*ccn1* y = −0.262x + 10.90, ZIKV RNA y = −0.243x + 10.42. (c) HEK293T cells were grown to 40–50% confluence in 12-well plates and co-transfected with 1500 ng of pCAGGS-Flag-NS, 250 ng of p-2299, and 50 ng of pRL-TK using ProFection calcium phosphate reagents. Subsequently, 24–48 h post-transfection, the cells were collected for luciferase detection. (d) Diagram of series CCN1 promoter mutant containing various *cis*-elements. (e) HEK293T cells were grown in 12-well plates and co-transfected with 1500 ng of pCAGGS-Flag-NS3, 250 ng of p-2299, p-2001, p-895, p-409, p-40 or p-CRE-mut, and 50 ng of pRL-TK using ProFection calcium phosphate reagents. Subsequently, 24–48 h post-transfection, the cells were collected for luciferase detection. (f) CCF-STTG1 cells were transfected with 2000 ng Flag-NS3 per well in a 6-well plate using Lipofectamine^TM^ 3000. The cells were subsequently harvested for western blot at 24 h post-transfection.The relative p-CREB and CCN1 protein levelin three experiments were summarized in the bar graph on the right. Each experiment was repeated three times. Statistical analyses were performed using GraphPad Prism 6.0 software. Data are expressed as the mean ± standard deviation (SD). (*, P < 0.05; ***, P < 0.001).

To investigate whether viral invasion or viral replication could induce increased CCN1 expression, we used UV inactivated ZIKV UV-ZIKV in infection assays. To inactivate ZIKV, we used direct UV irradiation, a conventional method used to inactivate viruses [42]. After 30 min of UV irradiation, UV-ZIKV was used to infect CCF-STTG1 cells and ZIKV-E protein was not detected 24 h post-infection (Figure 4(a)). However, ZIKV RNA was detected 12 h post-infection in the UV-ZIKV group, but the amount was much lower than that of the ZIKV group (Figure 4(b)), indicating that the virus had lost its ability to replicate. This indicates that UV-ZIKV was effectively inactivated. Compared to findings by the mock group and ZIKV group, enhancement of CCN1 expression was not observed in the UV-ZIKV group (Figure 4(a,b)). This suggests that ZIKV replication is required to stimulate CCN1 expression.

To investigate which nonstructural proteins are involved in the regulation of CCN1 expression, we used the dual luciferase reporter system. Different nonstructural protein expression plasmids were transiently transfected into HEK293T cells with the full-length CCN1 promoter (p-2299) and TK plasmids. The expression of nonstructural proteins was detected using western blot. The results showed that NS3 could significantly enhance the activation of the full-length CCN1 promoter (Figure 4(c)).

To identify the CCN1 promoter elements affected by NS3, promoter reporter,plasmids containing different elements were constructed (Figure 4(d)). The results showed that promoter activation was lost when the promoter was deficient in the CRE element, suggesting that the CRE of CCN1 is the key target element of NS3 (Figure 4(e)).

To determine whether CREB, the nuclear transcription factor of CRE, was activated by NS3, we harvested Flag-NS3-transfected CCF-STTG1 cells 24 h post-transfection for western blot. The results demonstrated that NS3 enhanced the phosphorylation of CREB at Ser133as well as CCN1 expression (figure 4(f)).

### CREB phosphorylation affects CCN1 expression in ZIKV infection

**Figure 5.**
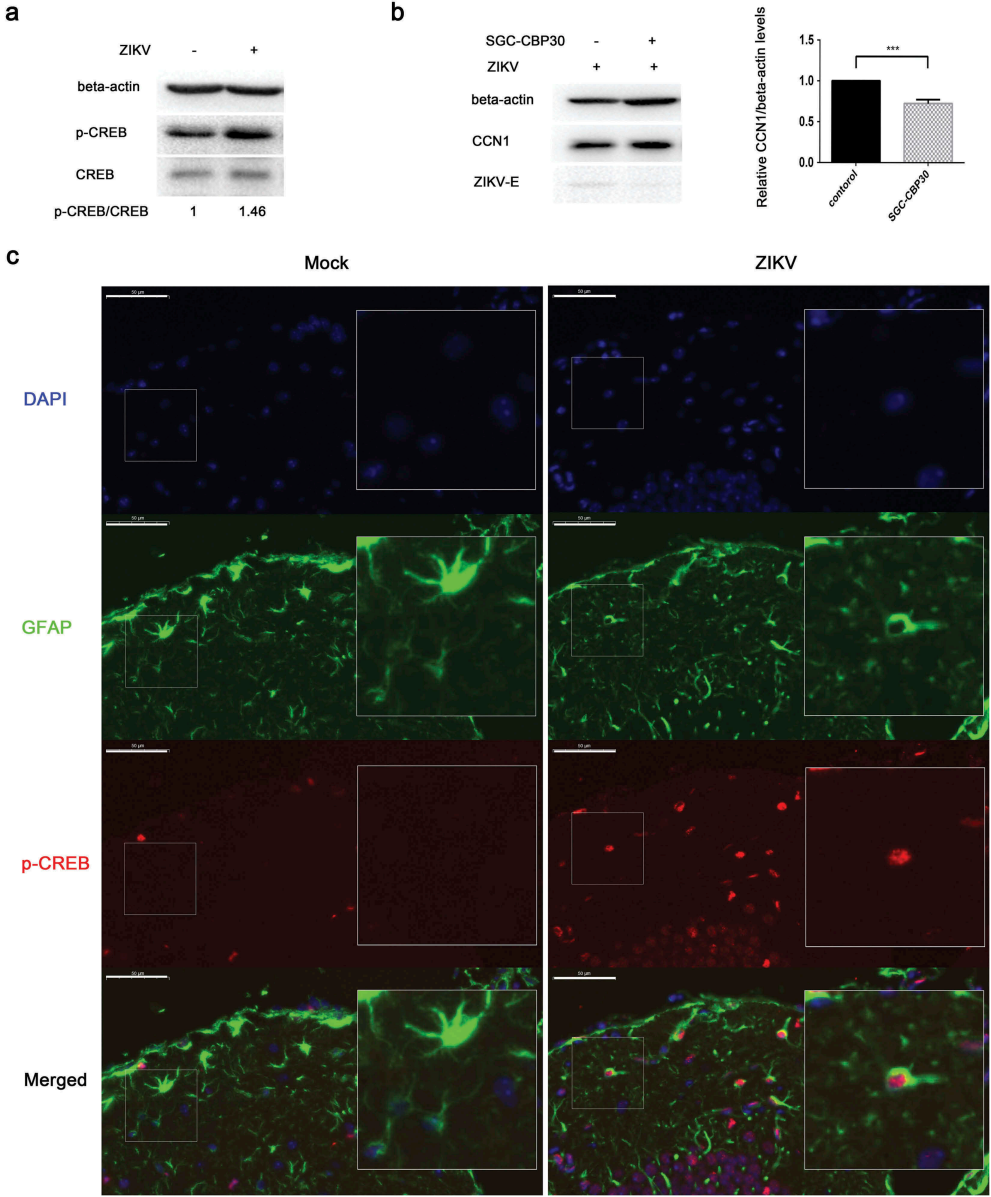
Phosphorylation of CREB Ser133 affects CCN1 expression and ZIKV replication. (a) CCF-STTG1 cells were infected with ZIKV at MOI = 3 TCID_50_/celland harvested 6 h post-infection for western blot. Densitometry ratioofp-CREB blots toCREB were labeled below the blots (b) CCF-STTG1 cells were infected with ZIKV in the presence of the inhibitor SGC-CBP30 (1 µM), washed with 1× PBS, and placed in RPMI-1640 medium with 10% FBS containing 1-µM SGC-CBP30. The cells were harvested 24 h post-infection for western blot. The relative CCN1 protein level in three experiments were summarized in the bar graph on the right and expressed as mean ± S.D (c) On day 5 post-infection, the brain was stripped and analyzed by immunofluorescence staining. GFAP (green), p-CREB (red), DAPI (blue). (Bar = 50 μm). Each experiment was repeated three times. Statistical analyses were performed using GraphPad Prism 6.0 software. Data are expressed as the mean ± standard deviation (SD). (***, P < 0.001).

Sendai virus (SV) mediates the activation of the transcription factor CREB [43]. To investigate whether CREB was activated during ZIKV infection thus leading to enhanced CCN1 protein expression, ZIKV-infected CCF-STTG1 cells were harvested 6 h post-infection. The level of CREB Ser133 phosphorylation was enhanced 6h post-infection (Figure 5(a)). Further experiments were performed using SGC-CBP30, a highly potent and selective CREBBP/EP300 bromodomain inhibitor. SGC-CBP30 binds CREB and acts as a scaffold to stabilize additional protein interactions with the transcription complex. SGC-CBP30 reduced the expression of CCN1 and ZIKV-E protein 24 h post-infection (Figure 5(b)). These results suggest that ZIKV infection in CCF-STTG1 cells activates the CCN1 protein via the CREB pathway.

Immunofluorescence results indicate that Ser133-phosphorylated CREB appeared primarily in the nucleus of GFAP-labeled astrocytes and that Ser133-phosphorylated CREB positive cells increased in the ZIKV group compared with those of the mock group (Figure 5(c)).

### NS3 binds to the regulatory domain of CaMKIIα to enhance phosphorylation at Thr286

**Figure 6.**
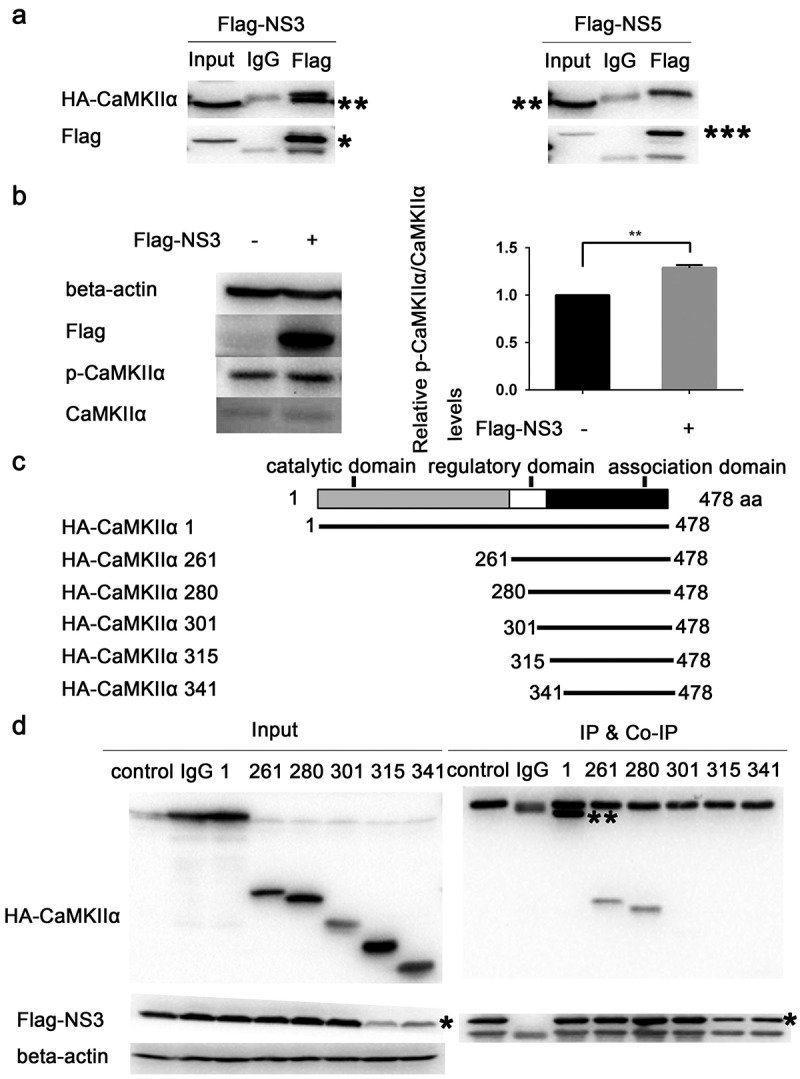
NS3 binds to the regulatory domain of CaMKIIα to enhance phosphorylation at Thr286. (a) HEK293T cells were co-transfected with 3000 ng HA-CaMKIIα and 3000 ng Flag-NS3 or Flag-NS5 in 60-mm dishes using ProFection calcium phosphate reagents. The cells were harvested for Co-IP experiments 28 h post-transfection. (b) CCF-STTG1 cells were transfected with 2000 ng Flag-NS3 per well in a 6-well plate using Lipofectamine^TM^ 3000. These cells were harvested for western blot 24 h post-transfection. The relative level of p-CaMKIIα in three experiments were summarized in the bar graph on the right and expressed as mean ± S.D . (c) The schematic map of a series of HA-CAMKIIα mutants was shown. (d) HEK293T cells were co-transfected with 1500 ng HA-CaMKIIα or CaMKIIα mutant and 1500 ng Flag-NS3 using ProFection calcium phosphate reagents. The cells were subsequently harvested for Co-IP experiments 28 h post-transfection (*, HA-CaMKIIα specific band; the other band is the antibody heavy chain). Control is pcDNA3.1(+), 1 is HA-CAMKIIα 1–478, 261 is HA-CAMKIIα mutant 261–478, 280 is HA-CAMKIIα mutant 280–478, 301 is HA-CAMKIIα mutant 301–478, 315 is HA-CAMKIIα mutant 315–478, 341 is HA-CAMKIIα mutant 341–478. Each experiment was repeated three times. (*, Flag-NS3 specific band; **, HA-CaMKIIα specific band; ***, Flag-NS3 specific band; the other band is the Flag antibody heavy chain).

Using tandem affinity purification (TAP) binding mass spectrometry, CaMKIIα was identified as a target protein that interacts with ZIKV NS3.The top-15 proteins with the highest possibility of interacting with ZIKV NS3 are listed in Table 2. The specific interaction of CaMKIIα with NS3 was further verified by Co-IP. HEK293T cells were co-transfected with HA-CaMKIIα and Flag-NS3 or Flag-NS5 and harvested for Co-IP assaywith anti-Flag antibodyat 28 h post-transfection. The results showed that CaMKII binds to NS3 but not to NS5 (Figure 6(a)), suggesting that the binding is specific. To verify the effect of NS3 on CaMKIIα activity, we examined the phosphorylation of CaMKIIα at Thr286 24h post-transfection. The results indicate that NS3 enhanced the phosphorylation of CaMKIIα at Thr286 (Figure 6(b)).

**Table 2.** Partial mass spectrometry results for TAP-tagged ZIKV NS3.

Number	Accession	Name
1	sp|P49327|FAS_HUMAN	Fatty acid synthase OS = Homo sapiens GN = FASN PE = 1 SV = 3
2	sp|Q92598|HS105_HUMAN	Heat shock protein 105 kDa OS = Homo sapiens GN = HSPH1 PE = 1 SV = 1
3	sp|P06576|ATPB_HUMAN	ATP synthase subunit beta, mitochondrial OS = Homo sapiens GN = ATP5B PE = 1 SV = 3
4	tr|B0QY89|B0QY89_HUMAN	Eukaryotic translation initiation factor 3 subunit L OS = Homo sapiens GN = EIF3L PE = 1 SV = 1
5	sp|P12004|PCNA_HUMAN	Proliferating cell nuclear antigen OS = Homo sapiens GN = PCNA PE = 1 SV = 1
6	tr|A0A087X2I1|A0A087X2I1_HUMAN	26S protease regulatory subunit 10B OS = Homo sapiens GN = PSMC6 PE = 1 SV = 1
7	sp|Q99714|HCD2_HUMAN	3-hydroxyacyl-CoA dehydrogenase type-2 OS = Homo sapiens GN = HSD17B10 PE = 1 SV = 3
8	tr|Q5H928|Q5H928_HUMAN	3-hydroxyacyl-CoA dehydrogenase type-2 OS = Homo sapiens GN = HSD17B10 PE = 1 SV = 1
9	sp|P09543|CN37_HUMAN	2ʹ,3ʹ-cyclic-nucleotide 3ʹ-phosphodiesterase OS = Homo sapiens GN = CNP PE = 1 SV = 2
10	tr|H0YBR5|H0YBR5_HUMAN	Eukaryotic translation initiation factor 3 subunit E (Fragment) OS = Homo sapiens GN = EIF3E PE = 1 SV = 1
11	sp|Q9UQM7|KCC2A_HUMAN	Calcium/calmodulin-dependent protein kinase type II subunit alpha OS = Homo sapiens GN = CAMK2A PE = 1 SV = 2
12	sp|P50851|LRBA_HUMAN	Lipopolysaccharide-responsive and beige-like anchor protein OS = Homo sapiens GN = LRBA PE = 1 SV = 4
13	tr|Q5T4U5|Q5T4U5_HUMAN	Acyl-Coenzyme A dehydrogenase, C-4 to C-12 straight chain, isoform CRA_a OS = Homo sapiens GN = ACADM PE = 1 SV = 1
14	sp|P11310|ACADM_HUMAN	Medium-chain specific acyl-CoA dehydrogenase, mitochondrial OS = Homo sapiens GN = ACADM PE = 1 SV = 1
15	sp|Q9UNY4|TTF2_HUMAN	Transcription termination factor 2 OS = Homo sapiens GN = TTF2 PE = 1 SV = 2

CaMKIIαis composed of an N-terminal catalytic domain (residues 1–260), a regulatory domain (residues 261–309), and a C-terminal association domain (residues 310–478) [44]. To determine the binding site of NS3 on CaMKIIα, aseries of CaMKIIα mutant plasmids, including 261–478, 280–478, 301–478, 315–478, and 341–478 residues, were constructed for the Co-IP assay (Figure 6(c)). The results showed that residues 261–478 and 280–478 but not residues 301–478, 315–478, and 341–478 interacted with NS3 (Figure 6(d)). This suggests that the NS3 interaction site is located between residues 280 and 300, which is within the regulatory domain of CaMKIIα. It is noteworthy that the interaction between mutant 1–478 and NS3 is much stronger than that between mutants 261–478 and 280–478, indicating an additional interaction between NS3 and the catalytic domain in addition to the regulatory domain (Figure 6(d)).

### NS3 in cooperation with CaMKIIα activates CRE and up-regulates CCN1 transcription

**Figure 7.**
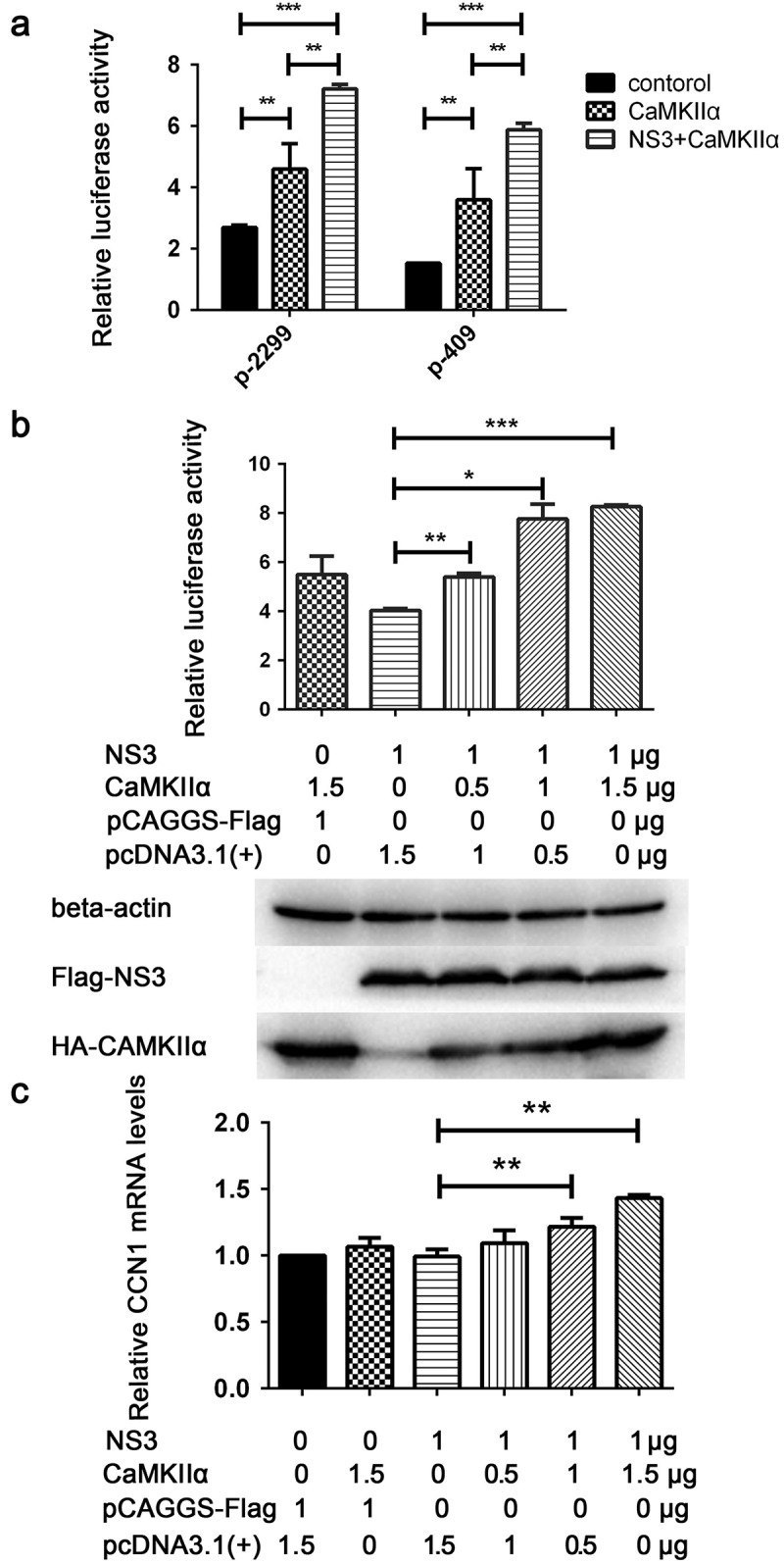
NS3 in cooperation with CaMKIIα activates CRE and up-regulates CCN1 transcription. (a) The pcDNA3.1(+) (900 ng) and pCAGGS-Flag (800 ng) (as a control) or HA-CaMKIIα (900 ng) and Flag-NS3 (800 ng) or HA-CaMKIIα (900 ng) and pCAGGS-Flag (800 ng), together with pRL-TK, and p-2299 or p-409 were co-transfected into HEK293T cells using ProFection calcium phosphate reagents. Subsequently, 48 h post-transfection, double luciferase assays were performed. The results were presented as Relative Luciferase Activity (firefly luciferase activity/renilla luciferase activity). (b) Various doses of HA-CaMKIIα, 1000-ng Flag-NS3, p-409, and pRL-TK were transfected into HEK293T cells using ProFection calcium phosphate reagents. The cells were harvested 24 h post-transfection for dual reporter luciferase assays. The results were presented as Relative Luciferase Activity (firefly luciferase activity/renilla luciferase activity). (c) Various doses of HA-CaMKIIα and 1000-ng Flag-NS3 were used for transient transfection into CCF-STTG1 cells using Lipofectamine^TM^ 3000 reagent. The cells were harvested 24 h post-transfection for RT-qPCR (comparative delta–delta Ct). Each experiment was repeated three times. Statistical analyses were performed using GraphPad Prism 6.0 software. Data are expressed as the mean ± standard deviation (SD)(*, P < 0.05; **, P < 0.01; ***, P < 0.001).

It has been reported that CaMKII can activate the CRE of a target gene promoter via CREB phosphorylation [45,46]. To determine whether CaMKIIα or the combination of CaMKIIα with NS3 can activatethe CRE of the CCN1 promoter, CaMKIIα and NS3 expression plasmids, pRL-TK, and p-2299 or p-409 were co-transfected into HEK293T cells. Subsequently, 48 h post-transfection, double luciferase assays were performed. Compared with control group transfected pCAGGS-flag and pcDNA3.1, CaMKIIα activated both the full-length promoter p-2299 and the mutant promoter p-409 containing only the CRE, whereas NS3 appeared to enhance this activation (Figure 7(a)). Further experiments were performed to assess whether the interaction of NS3 with CaMKIIα was responsible for the enhanced CCN1 CRE activation. Various amounts of CaMKIIα expression plasmids together with NS3, pRL-TK, and p-409 were co-transfected into HEK293T cells. The cells were harvested 24 h post-transfection for dual reporter luciferase assays. The results showed that the enhanced activation of CCN1 CRE in the NS3&CaMKIIα groups was positively correlated with the amount of transfected CaMKIIα expressing plasmids (Figure 7(b)). Subsequently, the amount of CCN1 mRNA24 h post-transfection was detected by RT-qPCR. The results showed that, compared with the NS3 or CaMKIIα groups, the expression levels of CCN1 mRNA in the NS3&CaMKIIα groups were positively correlated with the transfection dose of the HA-CaMKIIα-expressing plasmids (Figure 7(c)). This demonstrates that NS3 cooperates with CaMKIIα to up-regulate CCN1 expression through the CRE on the CCN1 promoter.

### CaMKIIα enhances CREB phosphorylation, CCN1 expression, and ZIKV replication

**Figure 8.**
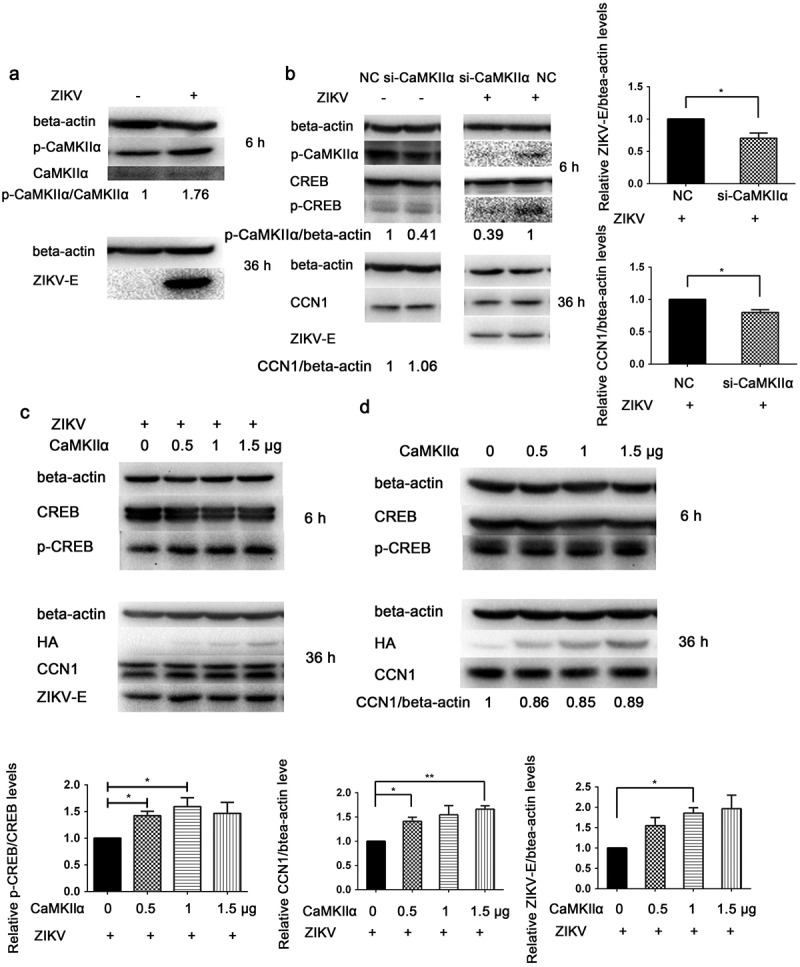
CaMKIIα regulates CREB Ser133 phosphorylation and affects CCN1 expression and ZIKV replication. (a) CCF-STTG1 cells were seeded in 35-mm dishes. After 16–18 h of culture, the cells were infected with ZIKV (MOI = 3) for 2 h. The cells were harvested 6 h or 36 h post-infection for western blot. Densitometry ratio of p-CaMKIIa blots to CaMKIIa were labeled below the blots (b) si-CaMKIIα (50-nM per well) was transfected into CCF-STTG1 cells in a 6-well plate using Lipofectamine^TM^ 3000. After transfection for 43–46 h, the cells were inoculated with RPMI or ZIKV (MOI = 3). Subsequently, the cells were harvested 6 h or 24 h post-infection for western blot. Densitometry from p-CaMKIIa and CCN1 blots normalized for beta-actin were labeled below the blots.The relative level of ZKIV E and CCN1 protein of three experiments were quantized and summarized in the bar graph on the right(c, d) HA-CaMKIIα (0–1.5-μg) per well was transfected into CCF-STTG1 cells in a 6-well plate using Lipofectamine^TM^ 3000. After transfection for 8–10 h, the cells were inoculated with ZIKV (MOI = 3) (c) or mock infected with RPMI-1640 (d) . Subsequently, the cells were harvested 6 h or 36 h post-infection for western blot. The relative level of p-CREB, CCN1 and ZKIV E protein of three experiments were quantized and summarized in the bar graphs below.The blots were visualized and analyzed using the Bio-Rad imaging system. Statistical analyses were performed using GraphPad Prism 6.0 software. Data are expressed as the mean ± standard deviation (SD) (*, P < 0.05; **, P < 0.01; ***, P < 0.001).

When CCF-STTG1 cells were infected with ZIKV, enhanced phosphorylation of CaMKIIα at Thr286 was detected (Figure 8(a)). When the ZIKV-infected CCF-STTG1 cells were transfected with NC or si-CaMKIIα, the phosphorylation level of CaMKIIα Thr286 and CREBSer133 decreased and the expressions of CCN1 and ZIKV-E protein reduced as well. Meanwhile, in uninfected cells, si-CaMKIIα does not significantly affect the CCN1 expression compared with the negative control (NC) (Figure 8(b)). These results indicate that, only in ZIKV infection, a decreased CaMKIIα can reduce the phosphorylation of CREB at Ser133, CCN1 expression, and virus replication. When the HA-CaMKIIα-transfected CCF-STTG1 cells were infected with ZIKV, the phosphorylation of CREB Ser133 showed an increasing trend that correlated with the transfection dose of HA-CaMKIIα plasmids; moreover, a unique up-regulating trend was observed for the expression of CCN1 protein.The ZIKV-E protein shows an increasing trend as well (Figure 8(c)). However, the expression of CCN1 was not affected in the uninfected cells (Figure 8(d)). These results suggest that, in ZIKV infection, CaMKIIα has a distinctive regulatory role in CREB activation and CCN1 expression that facilitates virus replication. Therefore,CaMKIIα is critical in ZIKV-enhanced CREB phosphorylation, CCN1 expression, and ZIKV replication.

## Discussion

As a pro-inflammatory factor, CCN1 expression can be enhanced by CVB3, oncolytic herpes simplex virus-1(HSV-1)and influenza A virus (IAV) infection [25,26,47]. In this study, we found that ZIKV also enhanced the expression of CCN1 in the astroglioma cell line CCF-STTG1. Furthermore, this enhancement was observed in the astrocytes of AG6 mice, a well-known model of ZIKV infection [48]. Astrocytes are an important type of glial cell and are critical for brain development and function [49]. Additionally, astrocytes participate in regulating synapse formation and immune responses in the brain [6,10]. Therefore, regulatory changes in the inflammatory factor CCN1 secreted by astrocytes and their role during neurotropic virus infection were investigated.

It has been reported that CCN1 expression aids the replication of coxsackievirus B3 (CVB3) [25]. Our study showed that CCN1 promoted ZIKV replication in CCF-STTG1 cells. In the presence of an anti-CCN1 monoclonal antibody, ZIKV replication was reduced in a dose-dependent manner. These results suggest that CCN1 likely binds to receptors on the cell membrane and activates downstream pathways that regulate ZIKV replication. It has been reported that CCN1 can activate multiple signaling pathways via binding to different integrin receptors [50], such as PI3K/Akt/NF-кB [51], ERK1/ERK2 MAPK [52], β-catenin-TCF/Lef [53], and AP-1-dependent pathways [54]. Interestingly, the nonsecretory mutant of CCN1also promotes the replication of ZIKV; it has been suggested that other intercellular targets of viral replication may have existed. The downstream CCN1 pathways that regulate ZIKV replication is worthy of investigation.

Our study mainly focuses on the mechanism by which ZIKV enhances CCN1 expression in astrocytes. The CCN1 promoter contains a variety of *cis*-regulatory elements and is regulated by various factors including growth factors, hormones, and cytokines [55]. We constructed a reporter plasmid, p-2299, containing the full-length CCN1 promoter to screen for viral proteins that activate CCN1 expression. Our study showed that UV-ZIKV could not induce the enhancement of CCN1 expression in CCF-STTG1 cells, suggesting that structural proteins may not be involved in the regulation of CCN1 expression. Therefore, the regulatory ability of seven nonstructural proteins on CCN1 promoter p-2299 was studied. Our results showedthat NS3 was important in this process. NS3 protein is a multifunctional protein with serine protease, nucleoside-triphosphatase-dependent RNA helicase, adenosine triphosphatase, and RNA 5-triphosphatase activities; additionally, it is important for viral replication [56]. However, the NS3 protein can only function with the assistance of the NS2B protein [57]; therefore, the role of NS2B-NS3 in the expression of CCN1 cannot be excluded. Additionally, owing to the low expression level of NS2B, as shown in Figure 4(c), the possible role of NS2B in the regulation of CCN1 cannot be ruled out. Thus, whether viral proteins other than NS3 regulate CCN1 expression necessitates further study.

Previous studies have indicated that the promoter region of CCN1 contains multiple response elements, including CArG, FAP, Oct, TBE1&2, AP1, and CRE [58]. As shown in Figure 2(b,c), the CCN1 level varied in the mock group. This phenomenon has been reported in a previous study [58], and it may be related to the CArG element in the CCN1 promoter, which is regulated by the SRF serum response factor. When serum is gradually used in the culture medium, the activity of CCN1 promoter decreases and the expression of CCN1 promoter decreases gradually. However, the CRE regulatory element in the CCN1 promoter activated by ZIKV is enough to activate the expression. Other promoter mutant studies indicated that the CRE on the CCN1 promoter was regulated by NS3. CREB is a trans-acting factor of the CRE. CREB is activated through the phosphorylation of Ser133, which increases its association with CREB-binding proteins or other transcription coactivators that increase the rate of transcription [59]. In our study, the phosphorylation of CREB Ser133 was enhanced during ZIKV infection or NS3 overexpression. Furthermore, SGC-CBP30, a CREBBP/EP300 inhibitor, could inhibit the ZIKV-induced enhancement of CCN1 expression and ZIKV replication. This suggests that CREB phosphorylation and CREB binding to CREBBP/EP300 are critical to ZIKV-mediated CCN1 expressions in astrocytes. Moreover, the CREB Ser133 phosphorylation state is determined by the activation levels of CREB kinases such as PKA, RSK, calmodulin kinase, and MSK1/2 [60]. Thus, the signaling pathway used by NS3 to influence CREB Ser133 phosphorylation and CCN1 expression is worthy of investigation.

CaMKIIα, an important kinase in the Ca^2+^ signaling pathway, was found to bind to the ZIKV NS3 protein. Further studies indicated that NS3 specifically interacted with CaMKIIα and that NS3 was primarily bound to the CaMKIIα regulatory domain residues 280–300 and enhanced its phosphorylation at Thr286. In the resting state, the autoinhibitory subdomain of CaMKIIα binds to the catalytic domain, of which residues 297–300 occupy the substrate binding site of the catalytic domain and residues 282–294 bind to the ATP binding site of the catalytic domain, interfering with the binding of ATP and kinases and inhibiting kinase activity [61]. When Ca^2+^/CaM binds to the CaM binding site of the CaMKIIα regulatory domain, the catalytic domain of the kinase is released to bind the substrate and ATP, thus promoting the Thr286 site phosphorylation of the autoinhibitory subdomain [62]. When the binding between the autoinhibitory subdomain and catalytic domain was blocked by CaMKIIα Thr286 phosphorylation, CaMKIIα was still activated, regardless of Ca^2+^ [62]. We speculated that the interaction of CaMKIIα with NS3 can lead to the continuous activation of CaMKIIα in a Ca^2+^/CaM-independent manner. Studies of chronic neuropathic pain caused by nerve injury and inflammation have shown that activated CaMKIIα can enhance CREB activity in the cell nucleus, thereby altering the expression of CRE-containing genes [63,64]. Additionally, our study indicated that NS3 overexpression could enhance the phosphorylation of CREB Ser133 and that NS3 binding to CaMKIIα could better stimulate CCN1 CRE activation and enhance CCN1 transcription. Therefore, NS3 could indeed affect CCN1 expression by activating CaMKIIα-CREB-CRE signaling cascade through interaction with CaMKIIα.

Furthermore, ZIKV could stimulate the phosphorylation of CaMKIIα Thr286. It has been reported that CaMKII activity is increased in nerve injury [65] and that CaMKII promotes inflammation [66]. The Thr286 phosphorylation site is known to be required for autonomous CaMKIIα activities [62]. In our study, the results of overexpression and interference demonstrate that CaMKIIα is involved in enhancing CREB Ser133 phosphorylation, CCN1 expression, and viral replication. Moreover, this regulation occurred in ZIKV infection. We hypothesize that NS3 is critical in activating the CaMKIIα-CREB-CCN1 signaling pathway by ZIKV to promote viral replication in astrocytes, which is beneficial to the spread of viruses in the CNS.

**Figure 9.**
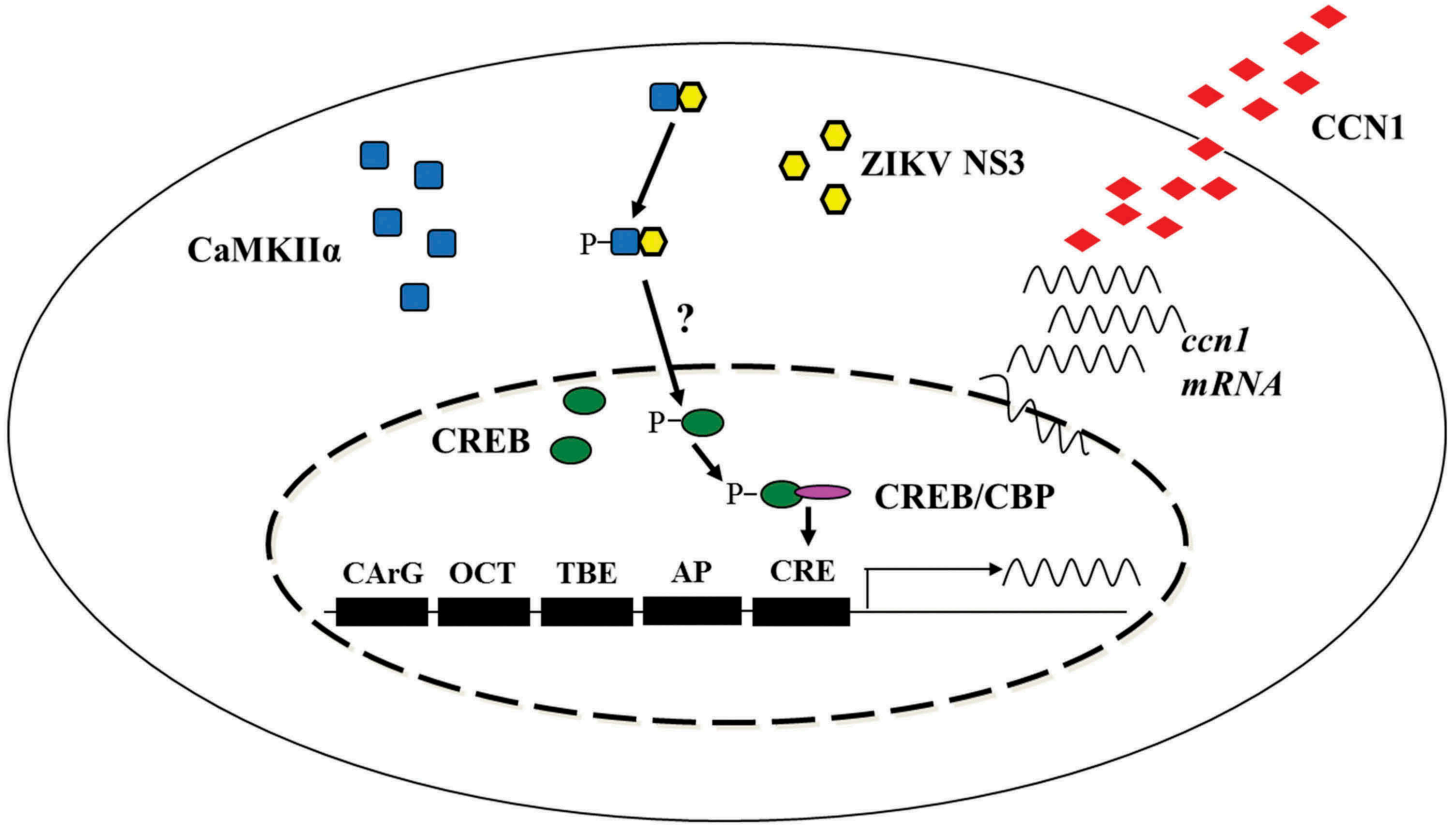
Schematic diagram of the mechanism by which ZIKV regulates CCN1 expression in astrocytes. We found that NS3 specifically interacted with the regulatory domain of CaMKIIα and enhanced CaMKIIα phosphorylation at the Thr286 site. Activated CaMKIIα enhanced CREB Ser133 phosphorylation activity in the cell nucleus, which increased its association with CBP or other transcription coactivators, thereby promoting the expression of CCN1 genes containing CRE. However, it is unclear whether CaMKIIα directly or indirectly regulates CREB phosphorylation.

In conclusion, we demonstrated that ZIKV infection up-regulates CCN1 protein in human astrocytes and that the NS3-CaMKIIα-CREB pathway is critical for CCN1 up-regulation (Figure 9). Moreover, our results suggest that the secretory CCN1 protein likely binds to receptors on the cell membrane to regulate ZIKV replication. Collectively, these findings elucidate the mechanisms by which ZIKV regulates CCN1 expression may provide information for the development of anti-ZIKV drugs.
